# Aspect Entropy Extraction Using Circular SAR Data and Scattering Anisotropy Analysis

**DOI:** 10.3390/s19020346

**Published:** 2019-01-16

**Authors:** Fei Teng, Wen Hong, Yun Lin

**Affiliations:** 1 Key Laboratory of Technology in Geospatial Information Processing and Application System, Chinese Academy of Sciences, Beijing 100190, China; scutengfei@163.com (F.T.); whong@mail.ie.ac.cn (W.H.); 2 Institute of Electronics, Chinese Academy of Sciences, Beijing 100190, China; 3 School of Electronic, Electrical and Communication Engineering, University of Chinese Academy of Sciences, Beijing 101408, China; 4 School of Electronic Information Engineering, North China University of Technology, Beijing 100144, China

**Keywords:** CSAR, anisotropy, aspect entropy, discrimination

## Abstract

In conventional synthetic aperture radar (SAR) working modes, targets are assumed isotropic because the viewing angle is small. However, most man-made targets are anisotropic. Therefore, anisotropy should be considered when the viewing angle is large. From another perspective, anisotropy is also a useful feature. Circular SAR (CSAR) can detect the scattering variation under different azimuthal look angles by a 360-degree observation. Different targets usually have varying degrees of anisotropy, which aids in target discrimination. However, there is no effective method to quantify the degree of anisotropy. In this paper, aspect entropy is presented as a descriptor of the scattering anisotropy. The range of aspect entropy is from 0 to 1, which corresponds to anisotropic to isotropic. First, the method proposed extracts aspect entropy at the pixel level. Since the aspect entropy of pixels can discriminate isotropic and anisotropic scattering, the method prescreens the target from the isotropic clutters. Next, the method extracts aspect entropy at the target level. The aspect entropy of targets can discriminate between different types of targets. Then, the effect of noise on aspect entropy extraction is analyzed and a denoising method is proposed. The Gotcha public release dataset, an X-band circular SAR data, is used to validate the method and the discrimination capability of aspect entropy.

## 1. Introduction

Synthetic aperture radar (SAR) is a high-resolution imaging radar that works all-weather and all-day [1]. SAR is widely used in military and civil fields because it assists in target analysis [2,3,4]. Scattering of a target is aspect-dependent. Therefore, targets are divided into two categories according to their scattering characteristic across azimuth: isotropic targets and anisotropic targets. In conventional SAR working modes, such as strip-map mode and spotlight mode, targets are assumed isotropic because the view angle is small. However, scattering variation cannot be ignored in some new SAR working modes. For example, circular SAR (CSAR) has a circular trajectory in order to observe the target at 360 degrees [5,6,7]. The aperture is so long that scattering differences in azimuth must be considered. The anisotropic scattering behavior is also a useful feature that can be obtained via CSAR. It can be used for target discrimination as man-made targets are usually anisotropic, while natural targets are usually isotropic. Additionally, different types of targets usually have varying degrees of anisotropy. 

Because anisotropic behavior has many uses, it has been widely studied in recent years [8,9,10]. Most of the studies are based on a polarimetric SAR system. Ferro-Famil et al. analyze the responses of anisotropic targets under different azimuth look angles [11,12]. Some researchers use polarimetric CSAR to obtain complete scattering information of targets. Xue et al. use polarimetric scattering entropy to analyze the anisotropic scattering [13]. Li et al. propose an anisotropic scattering detection method to characterize targets [14]. However, these methods all require the use of full-polarization data. In addition to the polarization characteristic, the scattering intensity is also aspect-dependent. Therefore, we can obtain the scattering behavior by using single-polarization CSAR data. Stojanovic et al. use the sub-aperture method to extract the curve of the radar cross section (RCS) amplitude of pixels versus aspect angles using single-polarization CSAR data [15]. The curve intuitively shows whether a target is anisotropic or isotropic. However, the curve is a high-dimensional feature that is not easy to use and the degree of anisotropy cannot be quantified by the curve. 

In this paper, we define aspect entropy as a descriptor of scattering anisotropy. Aspect entropy ranges from 0 to 1, which corresponds to anisotropic to isotropic. Our simulation results show the effectiveness of aspect entropy in quantifying the degree of anisotropy. As a result, we propose the extraction method of aspect entropy using real CSAR data. First, we propose the aspect entropy extraction method at the pixel level based on the sub-aperture method. Using the result of pixel-wise aspect entropy extraction, anisotropic pixels that belong to targets can be discriminated from isotropic clutters by thresholding. Next, we propose the aspect entropy extraction method at the target level. Thus, aspect entropy of targets can be extracted. The result can be used to analyze the scattering anisotropy of different targets and it has the capability of discrimination. During the aspect entropy extraction by using the real data, the RCS curve will have noise. Therefore, the effect of noise on aspect entropy extraction is studied. The simulation result shows that the aspect entropy is more accurate in high signal-to-noise ratio. Only high scatterings in the RCS curve is important on anisotropic target discrimination. Therefore, we proposed a RCS curve denoising method and it is shown effective by the simulation.

The Gotcha public release dataset is used to verify our aspect entropy extraction methods at the pixel and target levels. The result shows that the aspect entropy of pixels and targets can be extracted from CSAR data. Aspect entropy of pixels can be used to discriminate between isotropic and anisotropic scattering. The proposed RCS curve denoising method can remove the noise from the RCS curve extracted from the real data. It makes the result of aspect entropy extraction more accurate. Since the aspect entropy of different types of targets falls into different ranges, targets can be discriminated from each other by the aspect entropy value.

## 2. Concept of Aspect Entropy

Because the scattering of a target is aspect dependent, CSAR is helpful in detecting the anisotropic scattering behavior of a target. Radar cross section (RCS) is a measure representing the scattering ability of the incident electromagnetic wave [16]. RCS is related to the physical characteristics of the target and the parameters of the electromagnetic wave. Therefore, if the curve of the RCS amplitude versus the aspect angle (hereafter referred to as RCS curve) is known, we can see the scattering behavior of the target and judge whether a target is isotropic or anisotropic.

**Figure 1:**
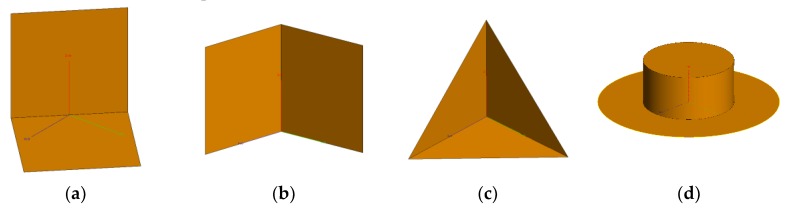
Models of canonical shapes. (**a**) Dihedral A. (**b**) Dihedral B. (**c**) Trihedral. (**d**) Top-hat.

**Figure 2:**
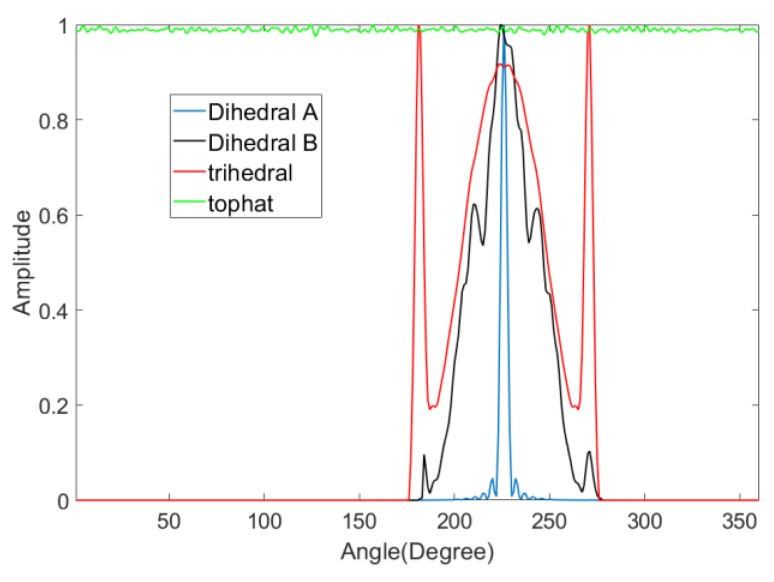
Radar cross section (RCS) curves of canonical shapes.

We can simulate the RCS curve for the selected shapes. The simulation is conducted at 10 GHz using vertical polarization with a 45° look-down angle. Four canonical shapes of the same size were used: a square plate dihedral set horizontally (named dihedral A), a square plate dihedral set vertically (named dihedral B), a triangular trihedral and a top-hat. The size is approximately 10 times longer than wavelength. They are all made of both perfect electric conductor and perfect magnetic conductor. The simulation is aimed at showing the ideal scattering mechanisms of these shapes. Therefore, these models are placed in the free space, whose relative permittivity is 1 and dielectric loss tangent is 0. The condition of the simulation is a more ideal than an anechoic chamber experiment. Figure 1 shows the models of the four canonical shapes. The results of the simulation are shown in Figure 2. The results show that the curve of the top-hat is smooth because it is isotropic. Since the dihedral and trihedral are both anisotropic, the high scatterings are concentrated in a limited range. However, we can discriminate between them by the degree of anisotropy, suggesting that different targets usually have different degrees of anisotropy. The scattering mechanism of dihedral A is very different from the trihedral. The scattering mechanism of dihedral B and trihedral are close but still discrepant. Targets can be discriminated if we find a method to quantify the anisotropy by a calculation. The calculation should be concerned with the scattering mechanism from different angles of view and not the RCS amplitude. 

Early physicists defined entropy as a measure of disorder. Entropy was introduced later in many other fields according to its application in physics. Shannon presented information entropy to describe the uncertainty of the information source [17]. Electromagnetism is the concept of polarization entropy, which quantifies the disorder of scattering [18]. Polarization entropy ranges from 0 to 1, which corresponds to zero scattering to perfect depolarizing. It is widely used in SAR image analysis [19,20]. The scattering in different aspect angles is similar to the scattering in different polarization types. Therefore, we present aspect entropy as a descriptor of scattering anisotropy. We can obtain the RCS amplitudes R(k) follow the angle θ(k) from curves as shown in Figure 2, where k=1,2,⋯,n. The pseudo-probability P(k) of scattering in θ(k) can be calculated by

(1) $$P(k)=\frac{R(k)}{\sum_{k=1}^{n}R(k)}.$$

Then, aspect entropy is defined as

(2) $$H_{a}=-\sum_{k=1}^{n}P(k)\log_{n}P(k).$$

$H_{a}$ ranges from 0 to 1. As shown in equation (2), $H_{a}$ is normalized by the sum of P(k) and is not concerned with RCS amplitude. Aspect entropy is inversely proportional to the pseudo-probability P(k). If the scattering is strong in some angles, the aspect entropy will be lower. Consequently, the aspect entropy of an anisotropic target is lower because the scattering of an anisotropic target at certain angles is much stronger than at other angles. The aspect entropy for an isotropic target is higher because the scattering is stable in all azimuth angles. We calculate the aspect entropy of the four models mentioned above and the results are listed in Table 1. The results show that aspect entropy can indicate the anisotropy diversity of the four shapes, and therefore can be used as a descriptor of anisotropy. The aspect entropy value of dihedral A and the top-hat have a large difference between these shapes. The scattering mechanisms of dihedral B and the trihedral are similar. It seems that the values are closed but the difference is big enough to discriminate them. The aspect entropy can still discriminate these two kinds of shapes by using the real data and the result is shown in Section 4.

## 3. Aspect Entropy Extraction

In this section, aspect entropy extraction methods at the pixel level and the target level are proposed respectively. Since the smallest unit of a CSAR image is a pixel, it is convenient to obtain the RCS curve of a pixel by using the sub-aperture method. Extracting the aspect entropy from pixels can help us analyze the scattering characteristics for the structure of a target and offer an overview of full-scene anisotropy. At the application level, targets are usually the object of analysis. Therefore, we propose the aspect entropy extraction method at the target level based on the aspect entropy of the pixels. The RCS curve of the target must be defined to calculate the aspect entropy. Targets of the same type are supposed to have similar aspect entropy, while the aspect entropy of different types of targets are diverse. Thus, targets can be discriminated from each other by using the aspect entropy value.

In addition, we study the effects of noise on aspect entropy extraction. Aspect entropy is more accurate in a high signal-to-noise ratio (SNR). Only high scatterings are interested in the RCS curve of the anisotropic target. Therefore, we can remove the noise out of the RCS curve. Then we propose a denoising method for the RCS curve. The result of simulations shows that aspect entropy is more accurate after denoising.

### 3.1. Aspect Entropy Extraction Method at the Pixel Level

**Figure 3:**
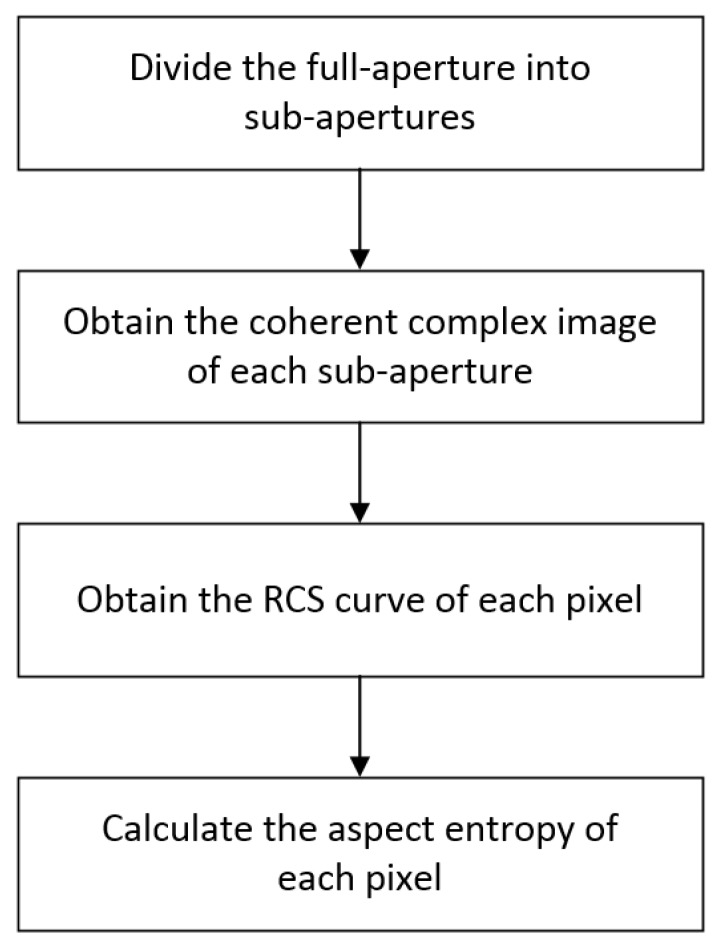
Flowchart of aspect entropy extraction at the pixel level.

In the SAR image, a pixel is the smallest unit, so it is reasonable to analyze the scattering anisotropy at the pixel level. The aspect entropy of pixels can be extracted by the method described below. A flowchart of the process is shown in Figure 3. First, the full-aperture is divided into sub-apertures. Second, the coherent complex image of each sub-aperture is obtained. Third, the absolute value of each pixel as the RCS amplitude is used to obtain the RCS curve of each pixel. Finally, the aspect entropy of each pixel is calculated. Details of the procedure are explained in the text below.

The first and second step establish the process for the sub-aperture method. The full-aperture is divided into k(k=1,2,⋯,n.) sub-apertures with the same width $\theta_{w}$. There is a trade-off when we choose the width $\theta_{w}$. The width must be large enough to obtain a high azimuth resolution. However, if the width is too large, the RCS amplitude will be inaccurate because the sub-aperture method uses the mean value of RCS amplitudes in $\theta_{w}$ as the RCS amplitude in the central angle θ(k). The coherent complex image of each sub-aperture is obtained by using the back projection (BP) algorithm [21,22]. When using the BP algorithm, the pixel size should be small enough to ensure that the scattering characteristic of each pixel is accurate. If the pixel size is too small, it will cause a substantial amount of computation. The pixel size can be set according to the theoretical resolution of the system. Then, we obtain the RCS curve through the 360° observation of each pixel. The coherent complex image $I_{n}(i,j)$ of each sub-aperture is the imaging result by using the BP algorithm. For each sub-aperture, the absolute pixel value of pixel (i,j) is used as the RCS amplitude $R_{ij}(k)$ in angle θ(k). For each pixel (i,j), the pseudo-probability $P_{ij}(k)$ of scattering in θ(k) can be obtained by Equation (1). The aspect entropy $H_{a}(i,j)$ of each pixel (i,j) can be calculated by Equation (2).

### 3.2. Aspect Entropy Extraction Method at the Target Level

**Figure 4:**
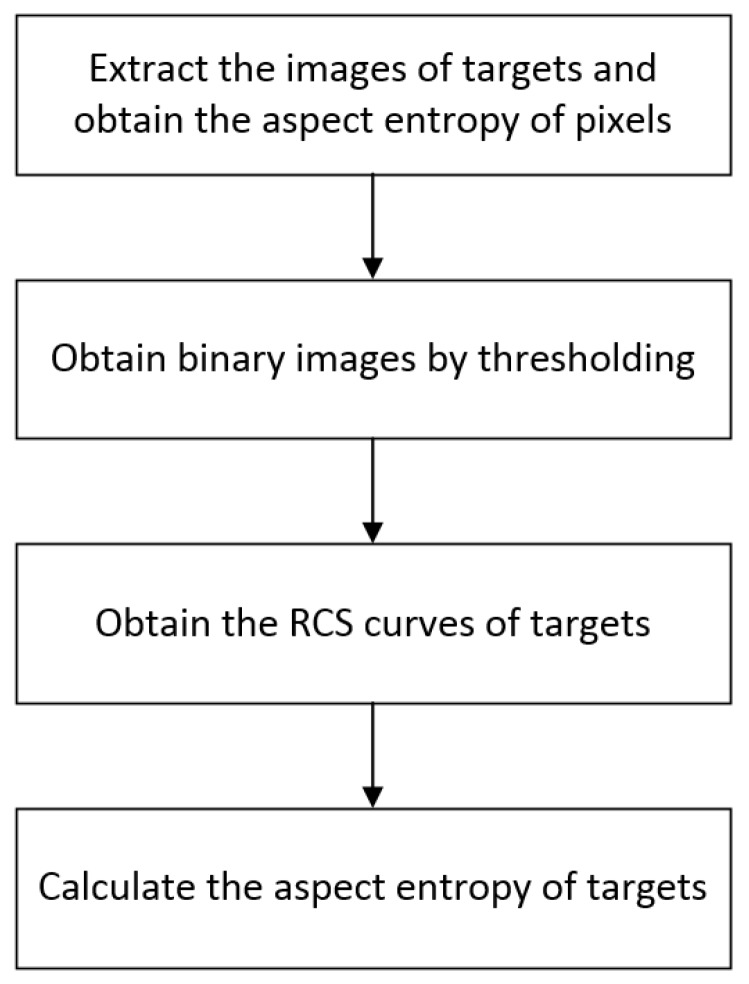
Flowchart of aspect entropy extraction at the target level.

Target discrimination and classification are important applications of SAR. The capability of aspect entropy on target discrimination is shown in Section 2. The aspect entropy of the target must be extracted to enable it to have a broader range of application. Usually, targets of interest are anisotropic because they contain many dihedral and trihedral structures. Natural clutters and man-made clutters such as lawns, trees, and roads are usually isotropic. Therefore, pixels from these clutters have a lower aspect entropy while pixels from the targets have a higher aspect entropy. Anisotropic pixels can be discriminated from isotropic pixels according to the aspect entropy value. We analyzed the scattering anisotropy of the targets by using the anisotropy of the pixels. To accomplish this, we propose the extraction method of aspect entropy at the target level. The process contains four steps and is described in Figure 4. First, the images of targets are extracted and the aspect entropy of the pixels is obtained. Second, the binary image is obtained by thresholding. Third, the RCS curves of the targets are obtained. Finally, the aspect entropy of each target is calculated.

The constant false-alarm rate (CFAR) or generalized likelihood ratio test (GLRT) [23,24] methods can be used to extract the targets from the image. For our purposes, the image of the target is extracted manually in this paper. The aspect entropy can be obtained using the same method described in subsection A. After thresholding according to the aspect entropy value, we can obtain a binary image. The value 1 represents anisotropic pixels, and 0 represents isotropic pixels. In the Circular SAR image, the pixel size is much smaller than the target size. A whole target or a structure of a target consists of many pixels. In the binary image, the targets consist of anisotropic pixels. Different types of targets are expected to have different aspect entropy. Therefore, we can analyze the scattering anisotropy of different types of targets using aspect entropy. Aspect entropy is calculated using the RCS curve as mentioned above. In subsection A, the RCS curve is associated with the pixel. Therefore, it is necessary to obtain the RCS curve of the target. If a target contains N anisotropic pixels after discrimination and the RCS curves of the pixels are $R_{m}(k)$ respectively, where m=1,2,⋯,N, we define the RCS curve of the target as

(3) $$R(k)=\sum_{m=1}^{N}R_{m}(k).$$

Scattering of the target at a certain angle is accomplished by the scattering of each pixel at this angle. Thus, the RCS curve of a target can be obtained by accumulation. This is equivalent to using a single pixel to represent the whole target. Next, we can calculate the aspect entropy of the target using (1) and (2). The aspect entropy is now applicable to the scattering anisotropy analysis at the target level.

### 3.3. Denoising of the RCS curve

When we extract the RCS curve by using the real data, the amplitudes are not 0 in angles which do not scatter the waves. These RCS amplitudes are regarded as noise in the RCS curves of targets. The noise mainly comes from the side lobes of clutters. During the coherent imaging process of a CSAR image, the value of a pixel from the image is actually affected by the side lobes of pixels around it. For example, a target pixel scatters the wave around θ and some clutters around this pixel scatter the wave in other angles. The RCS amplitude of this pixel is combined with its main lobe and the side lobes of the clutters. Consequently, isotropic targets are not influenced by the noise and clutters because these are isotropic too. While for an anisotropic target, the RCS amplitudes in other angles are not 0 in the RCS curve extracted by our method. The aspect entropy will become higher and inaccurate. Therefore, we have to denoise for the RCS curve of the anisotropic target.

**Figure 5:**
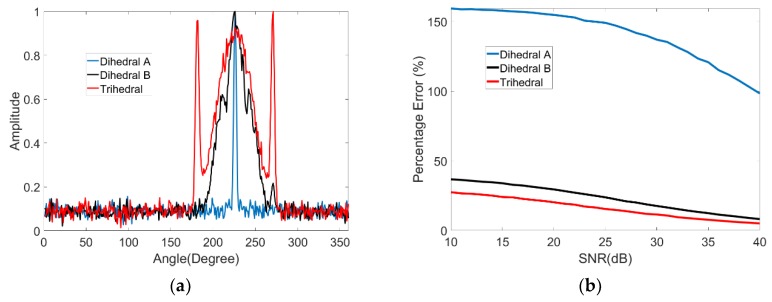
(**a**) RCS curves with noise (SNR = 20 dB). (**b**) Percentage error for aspect entropy calculation in different SNR.

Here, we define the SNR of the RCS curve as the ratio between the maximum power of the target and the power of the noise. To study the effect of the noise on the result of aspect entropy, we use the models mentioned in Section 2. Varying levels of noise are added into the RCS curve for the three anisotropic models: dihedral A, dihedral B, and the trihedral. The SNR ranges from 10 dB to 40 dB. As shown in Figure 5a, we choose SNR = 20 dB to illustrate the result of adding noise to the RCS curve. The percentage error for aspect entropy calculation in different SNR is shown in Figure 5b. For a certain anisotropic target, the higher the SNR is, the less the error is. With the same SNR, the percentage error is greater for targets that have higher degree of anisotropy. When we judge whether a target is anisotropic or not, we only interested in whether there are any high scatterings in some angles. Therefore, for anisotropic targets, the noise in other angles can be removed from the RCS curve. The easiest way to wipe out the noise is by setting a proper threshold value and eliminating as much noise as possible. The threshold value cannot be set directly. If we simply reset the amplitudes under a value to 0, lower scatterings belonging to targets would be removed too. Although these low scatterings are not the main lobe, they reflect the scattering characteristics of the target. Therefore, they should be protected during the denoising. The threshold value should be as close as possible to the ceiling of the noise.

**Figure 6:**
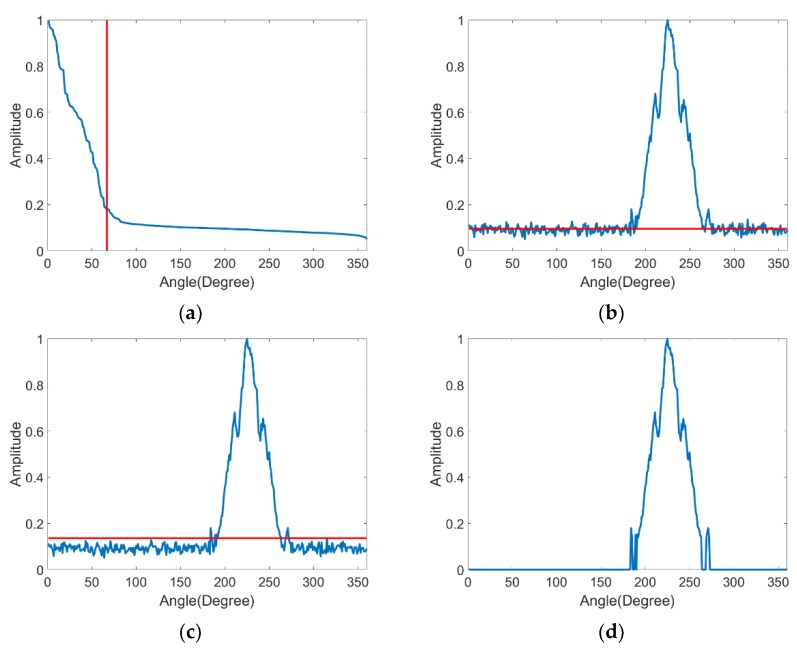
Diagram of the denoising procedure. (**a**) The result of sorting the RCS amplitudes into descending order and calculation of the energy concentration. (**b**) The result of mean value calculation for the noise. (**c**) The result of threshold value calculation. (**d**) The result of RCS curve denoising.

We proposed a denoising method for the RCS curve and Figure 6 is the diagram of the whole procedure. The RCS curve of dihedral B with SNR=20 dB is used as an example. To estimate the noise level of the RCS curve, the high scatterings should be removed. The energy concentration parameter W is defined as an approximation for the target high scattering persistence angle by Zhao et al. [25]. It is calculated by

(4) $$W=\frac{\sum_{k=1}^{n}R(k)}{R{\left(k\right)}_{\max}}.$$

First, calculate the energy concentration parameter of the RCS curve and round it up to an integer W. Sort the RCS amplitudes in descending order and remove the top W amplitudes. Figure 6a shows the result of sorting and the red line represents W=67. Second, calculate the mean value μ and the standard deviation σ for all remaining amplitudes, which are the noise. The red line shown in Figure 6b is μ=0.0954. Third, reset the RCS amplitudes under T=μ+2σ to 0. Here, we use double the standard deviation σ to ensure that the threshold value is higher than most of the noise. The red line shown in Figure 6c represents T=μ+2σ=0.1362. The result of RCS curve denoising is shown in Figure 6d. 

**Figure 7:**
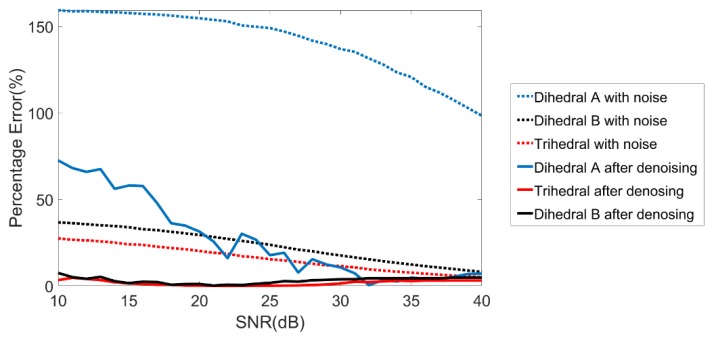
Comparison of the percentage error for aspect entropy calculation.

Figure 7 shows the comparison of the percentage error for aspect entropy calculation in different SNR between with and without denoising. It can be found that the denoising method significantly improved the accuracy of aspect entropy in low SNR. With the increase of the SNR, the percentage error after denoising has a small rebound, especially for the dihedral A. This is because the threshold value we set is higher than the ceiling of the noise to ensure that as much noise as possible can be removed. The level of the noise is very low when the SNR is high, so the threshold value can be higher than the low scatterings of the target. Therefore, the aspect entropy will be lower than the truth value. Although there is a rebound, the percentage error is still lower than it without denoising when the SNR is high. The simulation result shows that our denoising method is effective.

## 4. Experiment Results and Analysis

The Gotcha public release dataset was used to illustrate our method. Gotcha data consists of SAR phase history data collected at X-band with a 640 MHz bandwidth with full 360-degree azimuth coverage and full polarization [26]. There are many targets of interest in the imaging scene, including civilian vehicles, a top-hat, trihedrals, and dihedrals. 

### 4.1. Aspect Entropy Extraction at the Pixel Level

**Figure 8:**
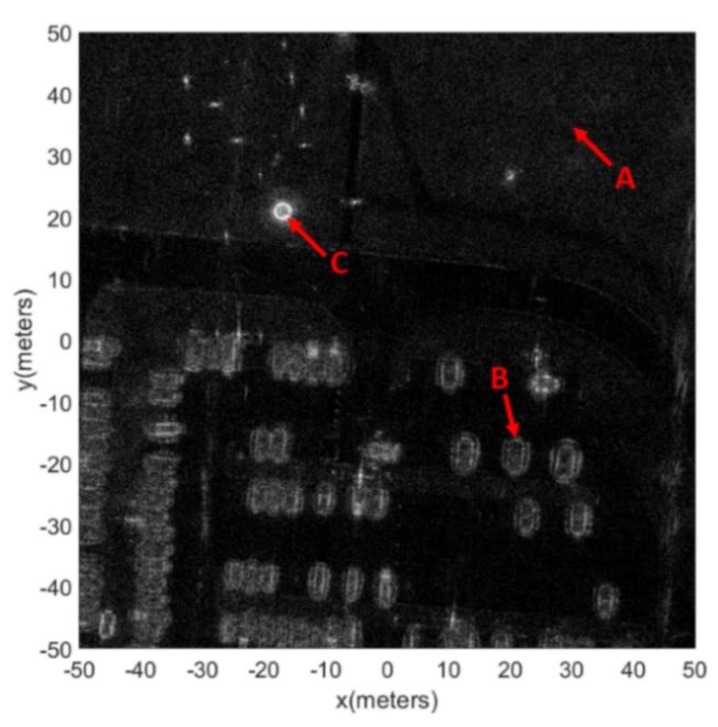
Coherent complex image of the full scene.

Our method uses only one polarization, and thus we select one pass and its’ HH polarization from the Gotcha data. Figure 8 is the coherent complex image of the full scene obtained by using the BP algorithm, and the pixel size is 0.025 m. 

**Figure 9:**
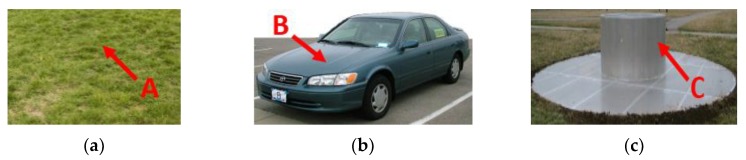
Optical images of three pixels. (**a**) Pixel A of the lawn. (**b**) Pixel B of a vehicle. (**c**) Pixel C of the top-hat.

**Figure 10:**
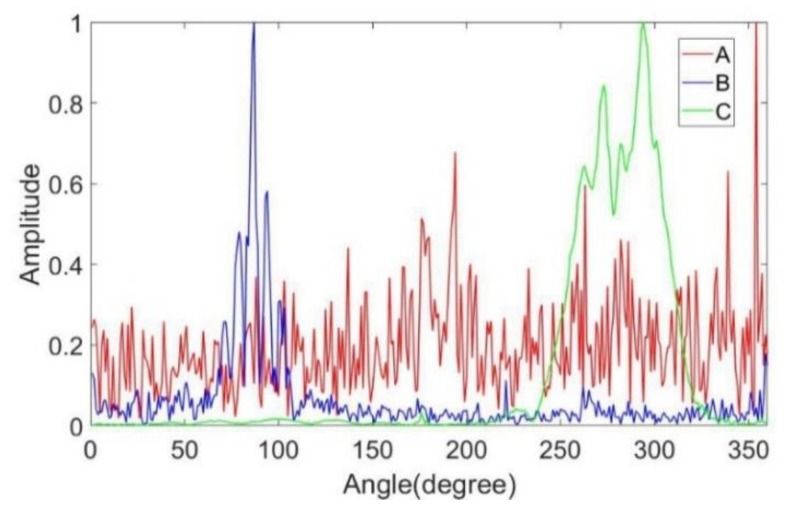
RCS curves of three pixels.

**Figure 11:**
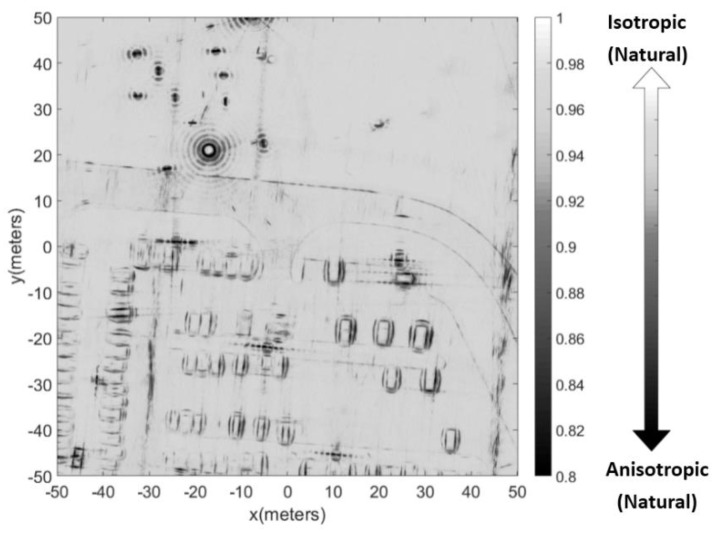
Aspect entropy image of the full scene.

When using the sub-aperture method to obtain the RCS curves, we found that using 360 sub-apertures offered a good compromise between the azimuth resolution and the preciseness of the RCS amplitude. As shown in Figure 8, three pixels were selected to show the result of the RCS curve extraction. Pixel A represents a pixel from the lawn. Pixel B represents a pixel from the frame of the vehicle. Pixel C represents a pixel from the edge of the top-hat. Figure 9 shows the optical images of the three targets. Figure 10 shows the RCS curves of the three pixels. Pixel A from the lawn is shown as isotropic scattering. Pixel B and pixel C are anisotropic because both are man-made metal structures. After obtaining the RCS curves of the pixels, the aspect entropy can be calculated. Figure 11 is the aspect entropy image of the full scene. The color bar indicates that darker colors denote a lower aspect entropy which means the pixels are more anisotropic, while the lighter color denotes a higher entropy and more isotropic. The result is as we expected. Pixels from vehicles and calibration targets show up as a dark color because they scatter the wave near a certain angle, which leads to a lower aspect entropy. Pixels from the lawn and roads show up in light color because they scatter the wave in all azimuth angles with similar intensity. Therefore, the aspect entropy can quantify the scattering anisotropy of pixels. Anisotropic scattering and isotropic scattering can be discriminated from each other in the aspect entropy image.

### 4.2. Aspect Entropy Extraction at the Target Level

**Figure 12:**
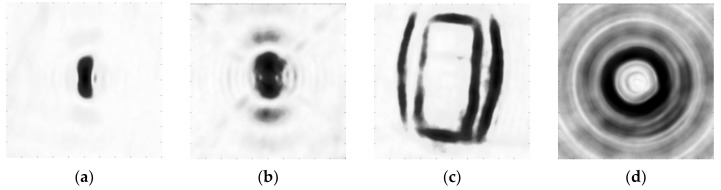
Aspect entropy images of targets. (**a**) Dihedral. (**b**) Trihedral. (**c**) Vehicle. (**d**) Top-hat.

**Figure 13:**
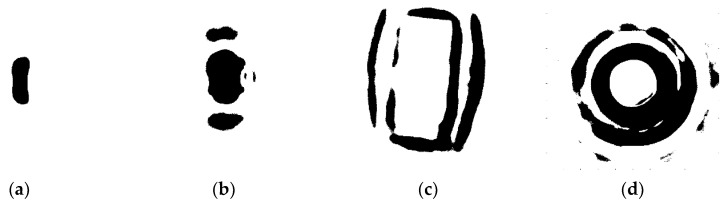
Binary images of the targets. (**a**) Dihedral. (**b**) Trihedral. (**c**) Vehicle. (**d**) Top-hat.

**Figure 14:**
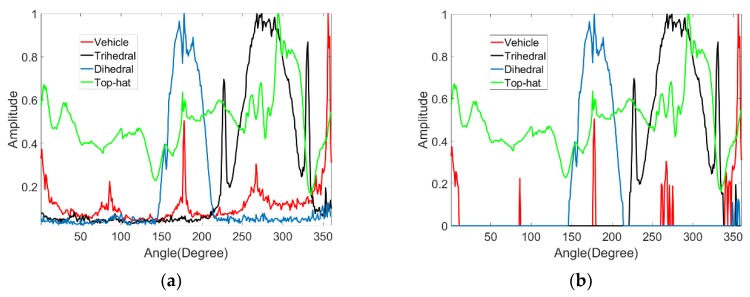
(**a**) RCS curves of the four targets. (**b**) RCS curves of the four targets after denoising.

We choose a dihedral, a trihedral, a vehicle, and a top-hat from the scene as examples to illustrate the procedure of target aspect entropy extraction. Figure 12 shows the aspect entropy images of the four targets. The results indicate that 0.91 is a suitable threshold value. Figure 13 shows the binary images of the four targets. According to Figure 13, anisotropic pixels from the targets can be segmented from isotropic clutters by thresholding. RCS curves of the four targets are obtained by using Equation (3), and the result is shown in Figure 14a. The RCS curves of the dihedral, trihedral, and the top-hat are similar to the result of the simulation discussed in Section 2. In addition, we can judge that the dihedral used in Gotcha experiment is set vertically because the RCS curve of it is the same as the curve of dihedral B in the simulation. The RCS curve of the vehicle is as expected. The four sides of the vehicle cause substantial scattering in four directions and barely any scattering in other directions. Unlike the RCS curves obtained by the simulation in Section 2, these RCS curves have the noise. Therefore, we use the method mentioned above to denoise the RCS curve. The parameters are $T_{1}=0.3$, k=  2. Figure 14b shows the RCS curves after denoising. It can be seen that most of the noise can be moved out and high scatterings are preserved well. The results show that our proposed aspect entropy extraction method can obtain the RCS curve of the target and extract the aspect entropy of the target. Our proposed RCS curve denoising method can preserve the useful information and obtain a good denoising effect. 

**Figure 15:**
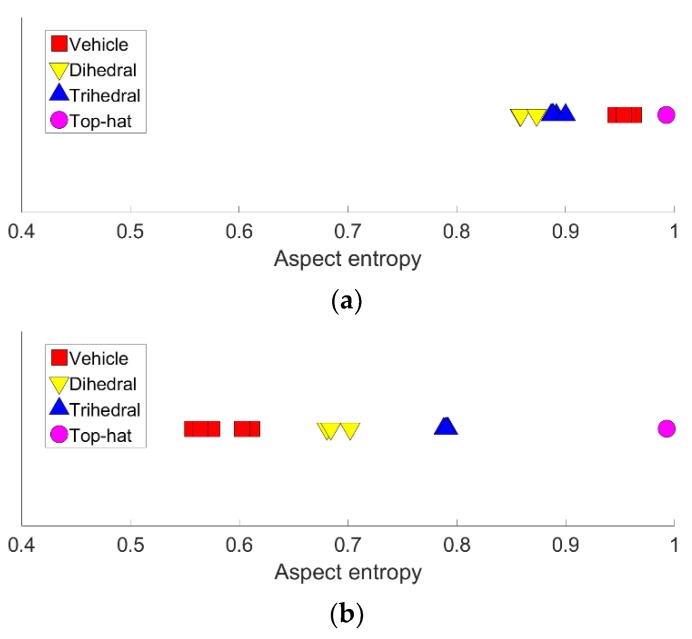
Visual result of discrimination. (**a**) Without denoising. (**b**) After denoising.

After obtaining the RCS curves of the targets, aspect entropy can be calculated using Equations (1) and (2). The full scene had a limited number of trihedrals and dihedrals that imaged clearly. To analyze the scattering anisotropy of the targets, six vehicles, six trihedrals, three dihedrals, and a top-hat were selected manually, and their aspect entropies were calculated. The locations and aspect entropy with and without denoising of the targets are listed in Table 2. The lower aspect entropy value represents the scattering of a target is more concentrated. Targets of the same type have similar aspect entropy values because they have the same scattering mechanism. The aspect entropy of dihedrals and trihedrals extracted from the real data without denoising is close to 1, which is much higher than the simulation results in Section 2. The aspect entropy after denoising is closer to the result of simulation and it has a greater ability of discrimination and clustering. The aspect entropy of targets of the same type is concentrated in a limited range. The aspect entropy of vehicles is in the range [0.5567, 0.6418]. The aspect entropy of trihedrals is in the range [0.7878, 0.7918]. The aspect entropy of dihedrals is in the range [0.6805, 0.7019]. The aspect entropy of the top-hat is 0.9927. In Figure 15, it is clear that vehicles, dihedrals, trihedrals, and the top-hat are clustered in different ranges. The ranges of different types are not coincident. The aspect entropy values of dihedral B and the trihedral are close in the simulation result so it is unclear whether the aspect entropy value can discriminate these two kinds of shapes in the real experiment. We set all the dihedrals as dihedral B in the Gotcha experiment. As can be seen in Figure 2 and Figure 14, the RCS curves of these two kinds of shapes are similar in both the simulation and the real experiment. The true scattering mechanisms of the targets can be restored well by the RCS curves after denoising. Thus, the aspect entropy values are close to the true value. In the experimental results, the aspect entropy values of dihedral are around 0.69 and the aspect entropy values of trihedral is around 0.79. Each kind of target was clustered well. Therefore, aspect entropy can discriminate between dihedral B and the trihedral despite their scattering mechanisms being similar. Therefore, different targets can be discriminated from each other according to the value of the aspect entropy. Beyond that, the RCS curve denoising greatly enhances the discrimination capabilities of aspect entropy.

The aspect entropy of the targets is affected by the frequency and polarization of the microwave. The aspect entropy of the targets is also significantly affected by the posture and size. Thus, the experimental results prove the capability of target discrimination in this X-band CSAR data and scene.

## 5. Conclusions

Scattering anisotropy analysis is important and useful in the field of SAR. Previous studies have mostly used polarimetric SAR data. In this paper, we use the CSAR data to analyze anisotropic scattering behavior across the azimuth. Aspect entropy is presented as a descriptor of scattering anisotropy, ranging from 0 to 1, which corresponds to anisotropic to isotropic. We verify that the aspect entropy can be the descriptor of scattering anisotropy by simulation. In addition, the effects of noise on the aspect entropy result is studied and a RCS curve denoising method is proposed. Aspect entropy extraction methods at the pixel and target level are respectively proposed using single-polarization CSAR data. The Gotcha public release dataset is used to illustrate our aspect entropy extraction methods. The results show that the aspect entropy of the pixel and the target can be successfully extracted by our methods. The value of the aspect entropy helps us to analyze the scattering anisotropy of the CSAR image. At the pixel level, aspect entropy can discriminate isotropic and anisotropic scattering. At the target level, it can discriminate different types of targets from each other. Further research will focus on the practical application of aspect entropy. It can be combined with other features and used for target detection or classification.

## Figures and Tables

**Table 1:** The aspect entropy of canonical shapes.

Shape	Aspect Entropy
Dihedral A	0.3823
Dihedral B	0.7131
Trihedral	0.7625
Top-hat	0.9999

**Table 2:** The aspect entropy of targets.

Target	Location	Aspect Entropy without Denoising	Aspect Entropy after Denoising
Chevy Malibu	(9.97, −5.22)	0.9469	0.5733
Ford Taurus Wag	(12.43, −18.21)	0.9468	0.5567
Toyota Camry	(20.66, −18.71)	0.9627	0.6118
Nissan Sentra	(31.42, −28.87)	0.9460	0.5753
Hyundai SantaFe	(22.68, −28.30)	0.9579	0.6024
Chevy Prizm	(35.44, −41.72)	0.9536	0.5639
Trihedral 1	(−24.39, 32.96)	0.8916	0.7918
Trihedral 2	(−32.50, 33.41)	0.8887	0.7895
Trihedral 3	(−32.14, 42.54)	0.8883	0.7915
Trihedral 4	(−28.09, 38.67)	0.8868	0.7878
Trihedral 5	(−13.86, 37.70)	0.8885	0.7918
Trihedral 6	(−5.12, 22.98)	0.9001	0.7907
Dihedral 1	(−15.55, 42.96)	0.8583	0.6805
Dihedral 2	(−18.58, 33.53)	0.8587	0.7019
Dihedral 3	(−26.15, 17.50)	0.8735	0.6841
Top-hat	(−17.00, 21.00)	0.9927	0.9927

## References

[1] Moreira A., Prats-Iraola P., Younis M., Krieger G., Hajnsek I., Papathanassiou K.P. (2013). A tutorial on synthetic aperture radar. IEEE Geosci. Remote Sens. Mag..

[2] Fornaro G., Reale D., Serafino F. (2009). Four-dimensional SAR imaging for height estimation and monitoring of single and double scatterers. IEEE Trans. Geosci. Remote Sens..

[3] Migliaccio M., Gambardella A., Tranfaglia M. (2007). SAR polarimetry to observe oil spills. IEEE Trans. Geosci. Remote Sens..

[4] Banerjee A., Burlina P., Chellappa R. (1999). Adaptive target detection in foliage-penetrating SAR images using alpha-stable models. IEEE Trans. Image Process..

[5] Lin Y., Hong W., Tan W., Wang Y., Xiang M. Airborne circular SAR imaging: Results at P-band. Proceedings of the 2012 IEEE International Geoscience and Remote Sensing Symposium.

[6] Dupuis X., Martineau P. Very high resolution circular SAR imaging at X band. Proceedings of the 2014 IEEE Geoscience and Remote Sensing Symposium.

[7] Chan T., Kuga Y., Ishimaru A. (1999). Experimental studies on circular SAR imaging in clutter using angular correlation function technique. IEEE Trans. Geosci. Remote Sens..

[8] Ponce O., Prats P., Pinheiro M., Rodriguez-Cassola M., Scheiber R., Reigber A., Moreira A. (2014). Fully polarimetric high-resolution 3-D imaging with circular SAR at L-band. IEEE Trans. Geosci. Remote Sens..

[9] Moses R., Potter L., Cetin M. (2004). Wide-angle SAR imaging. Proc. SPIE.

[10] Zhao Y., Lin Y., Hong W., Yu L. (2016). Adaptive imaging of anisotropic target based on circular-SAR. Electron. Lett..

[11] Ferro-Famil L., Reigber A., Pottier E., Boerner W.M. (2003). Scene characterization using subaperture polarimetric SAR data. IEEE Trans. Geosci. Remote Sens..

[12] Ferro-Famil L., Pottier E. Urban area remote sensing from L-band PolSAR data using time-frequency techniques. Proceedings of the 2007 Urban Remote Sensing Joint Event.

[13] Xue F., Lin Y., Hong W., Chen S., Shen W. (2018). An Improved H/α Unsupervised Classification Method for Circular PolSAR Images. IEEE Access.

[14] Li Y., Yin Q., Lin Y., Hong W. (2018). Anisotropy Scattering Detection from Multiaspect Signatures of Circular Polarimetric SAR. IEEE Geosci. Remote Sens. Lett..

[15] Stojanovic I., Cetin M., Karl W.C. (2008). Joint space aspect reconstruction of wide-angle SAR exploiting sparsity. Proc. SPIE.

[16] Odendaal J.W., Joubert J. (1996). Radar cross section measurements using near-field radar imaging. IEEE Trans. Instrum. Meas..

[17] Shannon C.E. (1948). A mathematical theory of communication. Bell Syst. Tech. J..

[18] Cloude S.R. (1995). Concept of polarization entropy in optical scattering. Opt. Eng..

[19] Shan Z., Wang C., Zhang H., An W. (2012). Improved four-component model-based target decomposition for polarimetric SAR data. IEEE Geosci. Remote Sens. Lett..

[20] Wakabayashi H., Matsuoka T., Nakamura K., Nishio F. (2004). Polarimetric characteristics of sea ice in the sea of Okhotsk observed by airborne L-band SAR. IEEE Trans. Geosci. Remote Sens..

[21] Ulander L.M.H., Hellsten H., Stenstrom G. (2003). Sythetic-aperture radar processing using fast factorized back-projection. IEEE Trans. Aerosp. Electron. Syst..

[22] Ponce O., Prats P., Rodriguez-Cassola M., Scheiber R., Reigber A. Processing of circular SAR trajectories with fast factorized back-projection. Proceedings of the 2011 IEEE International Geoscience and Remote Sensing Symposium.

[23] Kuttikkad S., Chellappa R. Non-Gaussian CFAR techniques for target detection in high resolution SAR images. Proceedings of the 1st International Conference on Image Processing.

[24] Lombardo P., Sciotti M., Kaplan L.M. SAR prescreening using both target and shadow information. Proceedings of the 2001 IEEE Radar Conference.

[25] Zhao Y., Lin Y., Hong W., Shen W., Xue F. Target aspect feature extraction and application from multi-aspect high resolution SAR. Proceedings of the 2017 IEEE International Geoscience and Remote Sensing Symposium (IGARSS).

[26] Ertin E., Austin C., Sharma S., Moses R., Potter L. (2007). GOTCHA experience report: Three-dimensional SAR imaging with complete circular apertures. Proc. SPIE.

